# Evaluation of syndromic algorithms for detecting patients with potentially transmissible infectious diseases based on computerised emergency-department data

**DOI:** 10.1186/1472-6947-13-101

**Published:** 2013-09-03

**Authors:** Solweig Gerbier-Colomban, Quentin Gicquel, Anne-Laure Millet, Christophe Riou, Jacqueline Grando, Stefan Darmoni, Véronique Potinet-Pagliaroli, Marie-Hélène Metzger

**Affiliations:** 1 Hospices Civils de Lyon, Hôpital de la Croix-Rousse, Unité d’hygiène et d’épidémiologie, F-69317 Lyon, France; 2 Université de Lyon, F-69000, Lyon; Université Lyon 1; CNRS, UMR5558, Laboratoire de Biométrie et Biologie Evolutive, F-69622 Villeurbanne, France; 3 Hospices Civils de Lyon, Direction Système d’Information et Informatique, F-69500 Bron, France; 4 Hospices Civils de Lyon, Pôle Information Médicale Evaluation Recherche, F-69424 Lyon, France; 5 Hospices Civils de Lyon, Hôpital de la Croix-Rousse, Service des urgences, F-69317 Lyon, France; 6 Hospices Civils de Lyon, Service d’hygiène et d’épidémiologie, F-69565 Saint-Genis-Laval, France; 7 CISMeF, LITIS EA 4108 - Université de Rouen, F-76031 Rouen cedex, France; 8 Service d’Hygiène, Épidémiologie et Prévention, Hôpital de la Croix-Rousse, 103, Grande-Rue de la Croix-Rousse, F-69317 Lyon Cedex 04, France

**Keywords:** Emergency service, Hospital, Syndromic surveillance, Detection algorithm, Infection control, Sensitivity and specificity, Population surveillance

## Abstract

**Background:**

The objective of this study was to ascertain the performance of syndromic algorithms for the early detection of patients in healthcare facilities who have potentially transmissible infectious diseases, using computerised emergency department (ED) data.

**Methods:**

A retrospective cohort in an 810-bed University of Lyon hospital in France was analysed. Adults who were admitted to the ED and hospitalised between June 1, 2007, and March 31, 2010 were included (N=10895). Different algorithms were built to detect patients with infectious respiratory, cutaneous or gastrointestinal syndromes. The performance parameters of these algorithms were assessed with regard to the capacity of our infection-control team to investigate the detected cases.

**Results:**

For respiratory syndromes, the sensitivity of the detection algorithms was 82.70%, and the specificity was 82.37%. For cutaneous syndromes, the sensitivity of the detection algorithms was 78.08%, and the specificity was 95.93%. For gastrointestinal syndromes, the sensitivity of the detection algorithms was 79.41%, and the specificity was 81.97%.

**Conclusions:**

This assessment permitted us to detect patients with potentially transmissible infectious diseases, while striking a reasonable balance between true positives and false positives, for both respiratory and cutaneous syndromes. The algorithms for gastrointestinal syndromes were not specific enough for routine use, because they generated a large number of false positives relative to the number of infected patients. Detection of patients with potentially transmissible infectious diseases will enable us to take precautions to prevent transmission as soon as these patients come in contact with healthcare facilities.

## Background

Patients who have potentially transmissible infectious diseases at the time of admission to healthcare facilities are a source of hospital-acquired infections. For example, studies on the incidence rates of diarrhoea, and particularly of acute viral gastroenteritis, recorded rates ranging from 0.15–19% in paediatric services [1-3]. The implementation of standard precautions and control measures after epidemic confirmation has proven efficiency in reducing rates of infection. Jusot et al. determined that some measures, e.g., “restricting the patient’s mobility outside his or her room, keeping the patient’s door closed, and having fewer than 20 beds in the ward,” were associated with lower rates of hospital-acquired diarrhoea in departments where they were applied [1].

A rapid and efficient warning system for early detection of patients with potentially transmissible infections who are admitted to hospital via the emergency department (ED) would facilitate prevention of transmission and deployment of control measures. Because the diagnosis of infections is not systematically implemented during ED visits, it is necessary to develop and adopt syndromic detection systems. Such a system would help infection control practitioners to work with clinicians in applying transmission-based precautions in a quick to react way.

There is little published literature evaluating syndromic surveillance systems intended to detect community-acquired transmissible infections among admitted patients [4]. Most syndromic surveillance systems that analyse ED data are designed to detect anomalous events occurring in the community. They provide syndrome classification by processing ED chief complaints [5-10] or ED discharge diagnoses [11,12]. These systems detect outbreaks efficiently because they consider a large amount of data to ascertain differences from baseline.

An automated clinical decision support system, aimed at detecting patients admitted to hospital with potentially transmissible infectious diseases, is being developed at Hôpital de la Croix-Rousse in Lyon (France). This system analyses computerised ED data entered in real time, not only chief complaints and discharge diagnoses but also clinical observations, specialists’ notes, prescriptions, etc. The system is based on the processing of structured and unstructured data from ED medical records. The technique of extraction and processing of textual data has already been described in a previous publication [13]. The objective of this new study, describing the stage after data processing, is to build and evaluate syndromic algorithms that use computerised ED data for early detection of patients with potentially transmissible infectious diseases. These algorithms will be direct applications of logistic models, using a common modelling strategy for different syndrome groups: respiratory system, gastrointestinal and cutaneous syndrome groups.

## Methods

### Setting and selection of patients

Syndromic algorithms to detect patients with potentially transmissible infectious diseases were built and evaluated in a retrospective cohort. The study population consisted of patients who were admitted to the ED and then hospitalised in Hôpital de la Croix-Rousse (University Hospital of Lyon, France) between June 1, 2007, and March 31, 2010. This hospital has 810 acute beds and exclusively manages adult patients. The total number of patients included in the cohort for this study was N=10,895.

### Infectious syndrome groups studied

The infectious disease studied in respiratory, cutaneous and gastrointestinal syndrome groups were defined based on the characteristics of the corresponding potentially transmissible infections. Syndromes selected for the study are those for which appropriate hygiene precautions should be applied to prevent the risk of transmission (contact precautions, droplet precautions and airborne-infection isolation precautions) [14]. Syndrome group of the respiratory system (abbreviated in the following text by “SGRS”) included lower-airway infections (e.g., pneumonia, influenza and influenza-like illness), upper-airway infections (e.g., pharyngitis) and tonsillitis. Tonsillitis was included in this group because transmission based precautions are common to other syndromes of the respiratory system (eg. viral tonsillitis, streptococcal tonsillitis). Then, it is interesting for the infection control practitioners, to also detect these infections. Cutaneous syndrome group included varicella-zoster, measles, rubella, scabies, erysipelas, suppurative abscess, cellulitis and phlegmons. Gastrointestinal syndrome group included viral gastroenteritis and diarrhoea due to bacterial infection (e.g., salmonella, dysentery and *Clostridium difficile*).

### Data collection and processing

In Hôpital de la Croix-Rousse, ED patient records are computerised. Clinical data consist of both structured variables (age, gender, discharge diagnostic code, etc.) and unstructured variables (chief complaints, clinical observations, etc.). Data stored daily in the hospital data warehouse were extracted by queries written by our Department of Information Systems, using Business Objects software.

The extracted data are:

– Identification number (anonymised for the study)

– Date and time of ED admission

– Date and time of ED discharge

– Residence postcode

– Age

– Gender

– Type of admission (e.g., spontaneous consultation, sent by family physician, brought by ambulance)

– Circumstances of admission

– CCMU code (French clinical classification of ED patients, Classification Clinique des Malades aux Urgences)

– Vital signs upon arrival (blood pressure, pulse, respiratory frequency, dyspnea, temperature, chills, purpura)

– Chief complaint*

– Clinical observation*

– Biological procedures*

– Technical diagnostic and therapeutic procedures*

– Type of imaging prescribed

– Type and free-text* of specialists’ notes

– Discharge diagnoses: ICD-10 codes and associated labels*

– Discharge prescriptions*

– Type of discharge (hospitalisation in the same hospital, hospitalisation in another hospital, discharge to home)

– Destination (department or hospital where the patient is hospitalised)

**Figure 1:**
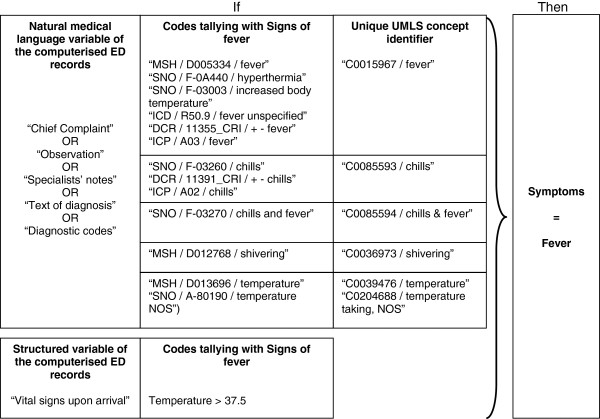
**Example of data processing for extracting the symptom “fever” from unstructured and structured variables in the medical record.** ICD, International Classification of Diseases, 10th revision (ICD-10); SNO, Systematized Nomenclature of Medicine, version 3.5 (SNOMED 3.5); MSH, Medical Subject Headings (MeSH); ICP, International Classification of Primary Care (ICPC-2); DCR, French Dictionary of Consultation Results (DCR); NOS, Not Otherwise Specified.

Natural medical-language variables (followed by * in the list above) need to be processed before their use. The method for doing so is described in another publication [13]. These variables were automatically processed using the UrgIndex application and the French-language medical multi-terminology indexer (French acronym, ECMT: Extracteur de Concepts Multi-Terminologique) [15]. Medical terms were coded according to standardised international terminologies. At the end of the automated process, only codes for infectious signs and symptoms were selected. Figure 1 shows an example of data processing for the symptom “fever,” including the coding process when it appears in natural language, in one of the variables in the medical record.

### Development method for building the detection algorithms

Data from the cohort (N=10,895) were divided into two datasets. The first dataset was constituted by randomly selecting 70% of the study population (N=7,627). This dataset was used for the training phase. A second dataset consisted of the remaining 30% of the study population (N=3,268) and was used to evaluate the performance of algorithms developed with the first dataset.

Separate detection algorithms were built for infectious respiratory, cutaneous and gastrointestinal syndrome groups, and each algorithm was separately assessed using the training set. To construct the algorithms, the signs and symptoms describing these syndromes, according to their locations in the electronic medical records, were fed into a logistic regression model. The 2 quantitative variables, age and number of inflammation signs in clinical notes, were categorized into 4 items for the logistic regression modelling. The algorithms were built independently for the different syndrome groups. Consequently, if a patient presented several infectious diseases corresponding to different syndrome groups, the patient’s data were used in the different models for building detection algorithms of each syndrome group. A descending procedure was performed to ascertain significant variables (p < 0.05), i.e., variables predictive of infection corresponding to the studied syndrome groups. The best logistic model was chosen for each syndrome group by including the variable set that yielded the lowest value of the Akaike information criterion (AIC). The corresponding individual probability of infection was calculated for each patient in the training set. The optimal threshold of detection was determined into two steps. In the first step, each of the individual probabilities calculated in the training set was used as the threshold of detection. The corresponding parameters of detection performance, sensitivity and specificity were calculated and plotted on receiver operating characteristic (ROC) curves. In the second step, the optimal threshold of detection was determined. For a sensitivity range between 75% (threshold for minimal acceptable sensitivity) and 100%, the number of true and false positives per week were estimated and plotted on curves. The optimal threshold corresponded to the individual probability giving a false positive/true positive ratio of 2 in the training set. The ratio of 2 for false positive/true positive was determined by consensus of our infection control team according to the weekly excess workload judged acceptable to investigate false positives, as compared to the work required to investigate true positives.

### Evaluation method of detection algorithms

The second dataset (test dataset) consisted of the remaining 30% of the study population (N=3,268). Detection of potentially infected patients in the test dataset used the following algorithm: 1) parameters estimated with the final model obtained using the logistic descending procedure in the training set were applied to the test dataset; 2) individual probabilities of infection were calculated; 3) when the individual probability was over the detection threshold, the patient was categorized as infected, otherwise the patient was categorized as not infected.

The detection algorithms were evaluated using a cross-validation procedure, and in terms of performance in detecting patients with infections corresponding to the syndrome group studied.

The cross-validation procedure consisted in comparing the distributions of individual probabilities between the training and test datasets by t-tests of the means and areas under the ROC curves.

The algorithms’ performances in detecting patients belonging to the SGRS, gastrointestinal syndrome group and cutaneous syndrome group were evaluated by calculating sensitivity, specificity and positive and negative predictive values (PPV and NPV). The reference used for the calculation of these parameters was the medical diagnosis of infection coded in the French Diagnosis-Related Group system (DRG; French acronym: PMSI). A patient was classified as having an infection corresponding to a respiratory, gastrointestinal or cutaneous syndrome group when the ICD-10 code was assigned in the French DRG by a physician during hospitalisation.

If the discharge summary did not contain an ICD-10 code corresponding to an infection whose symptoms were connected to SGRS, gastrointestinal or cutaneous syndrome group, the patient was classified as not having an infection corresponding to the syndrome group considered.

The 95% confidence intervals (CI) of sensitivity, specificity, PPV and NPV were computed by the exact binomial method. Analyses were undertaken with SAS 9.1 and R 2.8 softwares.

## Results

### Characteristics of the study population

The study population consisted of 10,895 adult patients who visited the ED and were then hospitalised in the same hospital between June 1, 2007, and March 31, 2010. The mean age of patients was 67.1 (±21.3) years. There were 5,136 men (47.1%).

The training dataset comprised 7,627 patients, including 713 (9.3%) patients with respiratory infections, 173 (2.3%) with cutaneous infections and 85 (1.1%) with gastrointestinal infections.

The test dataset consisted of 3,268 patients, including 318 (9.7%) patients with respiratory infections, 73 (2.2%) with cutaneous infections and 34 (1.0%) with gastrointestinal infections.

### Detection algorithms

#### Selection of the variables used in the detection algorithms

To calculate each individual probability of infection, the detection algorithms used the remaining variables in the model with the lowest AIC. The results are presented for respiratory, cutaneous and gastrointestinal syndrome groups in Tables 1, 2 and 3, respectively.

**Table 1:** Results of logistical regression procedures (final model) for respiratory syndromes (infected = 713; non-infected = 6,914)

**Effect**	**Odds ratio**	**95% Confidence interval**
Age ≤53 years	1	-
Age 54–74 years	1.63	1.21–2.2
Age 75–84 years	1.35	0.99–1.85
Age ≥85 years	1.16	0.85–1.60
Mention of diagnosis of respiratory infection in diagnosis section (ICD-10)	10.71	8.56–13.4
Mention of diagnosis in clinical notes	3.12	2.50–3.89
Cough in clinical notes	1.97	1.59–2.45
Sore throat in chief complaint	9.16	3.03–24.74
Abnormal pulmonary auscultation in clinical notes	1.54	1.22–1.94
Sign of respiratory failure in chief complaint	2.92	1.79–4.69
Sign of respiratory failure in clinical notes	1.93	1.57–2.36
Fever on observation	1.35	1.09–1.65
Microbiology examination in biological procedures	3.94	1.16–12.35
Biology examination in clinical notes	1.27	1.03–1.58
Biology examination in biological procedures	0.73	0.56–0.93

**Table 2:** Results of logistical regression procedures (final model) for cutaneous syndromes (infected = 173; non-infected = 7,454)

**Effect**	**Odds ratio**	**95% Confidence interval**
Mention of cutaneous infection in diagnosis section (ICD-10 codes)	38.37	21.87–68.54
Mention of cutaneous infection in chief complaint	5.44	2.65–11.15
Mention of cutaneous infection in clinical notes	6.29	3.92–10.04
Skin rash in clinical notes	2.89	1.14–6.73
Complication of skin infection in clinical notes	2.29	1.32–3.86
Number of inflammation signs in clinical notes = 0	1	-
Number of inflammation signs in clinical notes = 1	0.98	0.59–1.62
Number of inflammation signs in clinical notes = 2	2.18	1.15–4.06
Number of inflammation signs in clinical notes = 3	5.68	2.27–13.92
Fever in chief complaint	2.28	1.12–4.31
Biology examination in biological procedures	0.33	0.18–0.58
Microbiology examination in biological procedures	9.30	1.10–46.99
Opinion on infectious diseases reported in clinical notes	1.83	1.03–3.14
Specific treatment mentioned in clinical notes	2.67	1.58–4.41

**Table 3:** Results of logistical regression procedures (final model) for gastrointestinal syndromes (infected = 85, non-infected = 7,542)

**Effect**	**Odds ratio**	**95% Confidence interval**
Mention of gastrointestinal infection in diagnosis section (ICD-10 codes)	16.06	7.72–33.28
Mention of gastrointestinal infection in clinical notes	1.99	1.14–3.49
Diarrhoea in chief complaint	3.45	1.67–6.88
Diarrhoea in clinical notes	7.45	4.36–12.66
Fever in clinical notes	2.06	1.19–3.61
Biology examination in biological procedures	0.45	0.22–0.83
Microbiology examination in chief complaint	3.21	1.20–7.59
Specific treatment of gastrointestinal infection in therapeutic procedures	7.27	1.18–30.32

#### Determination of the optimal threshold of detection

**Figure 2:**
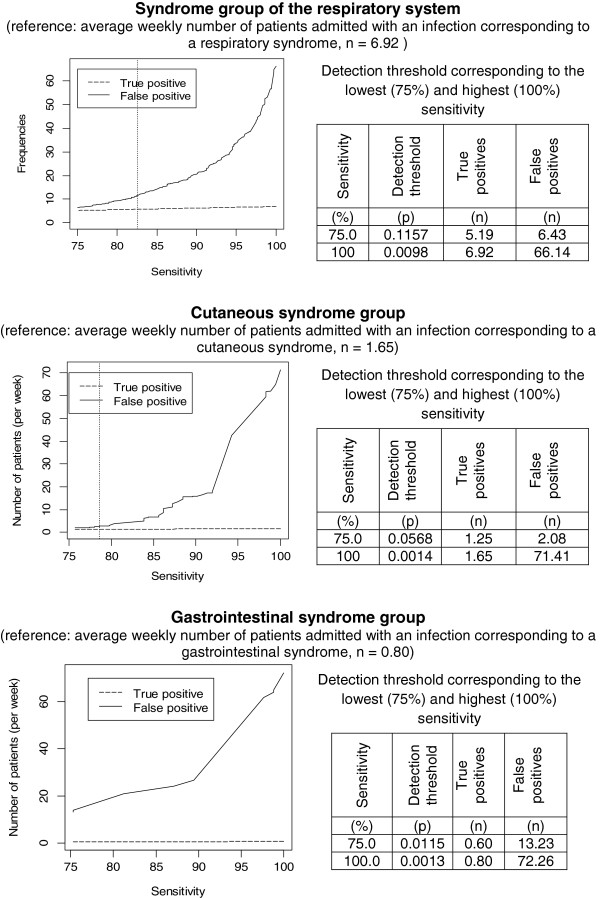
**Variation in the number of true and false positives per week according to sensitivity in the training set.** Numbers of true and false positives per week were calculated in the population studied (mean population per week=73.06; learning dataset). Tables show the values of optimal detection thresholds (vertical lines) and thresholds corresponding to 75% and 100% sensitivity.

The optimal threshold of detection was the individual probability corresponding to sensitivity greater than 75% and with a false positives/true positives ratio equal to 2 in the training set. Figure 2 shows the number of corresponding true and false positives for sensitivity between 75 and 100% in the training set (population studied = 7,627 patients). For SGRS, the number of false positives varied from 6.43 to 66.14 when sensitivity ranged from 75% (corresponding to a detection threshold set at an individual probability of 0.1157) to 100% (corresponding to a detection threshold set at an individual probability of 0.0098), corresponding to a range in the number of true positives from 5.19 to 6.92.

For cutaneous syndrome group, the number of false positives varied from 2.08 to 71.81 when sensitivity ranged from 75% (corresponding to a detection threshold set at an individual probability of 0.0568) to 100% (corre-sponding to a detection threshold set at an individual probability of 0.0014), corresponding to the range in the number of true positives from 1.25 to 1.65.

For gastrointestinal syndrome group, the number of false positives varied from 13.23 to 72.26 when sensitivity ranged from 75% (corresponding to a detection threshold set at an individual probability of 0.0115) to 100% (corresponding to a detection threshold set at an individual probability of 0.0013), corresponding to the range in the number of true positives from 0.60 to 0.80.

**Figure 3:**
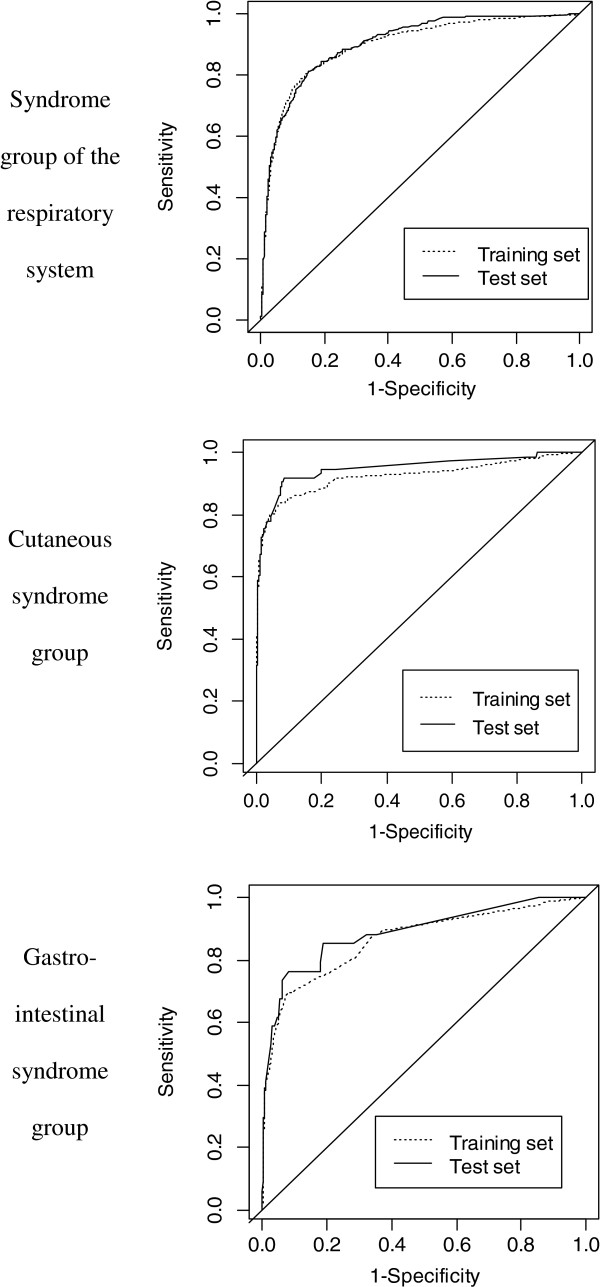
**Receiver operating characteristic (ROC) curves for detecting patients with potentially transmissible infectious diseases.** ROC curves were built by using each individual probability as the detection threshold of infection (training and test datasets were used separately).

ROC curves obtained with individual probabilities used as detection thresholds, generated separately for each studied syndrome group in the training set, are illustrated in Figure 3. For SGRS, the optimal probability threshold was 0.0661 (Figure 2). The corresponding expected number of infected patients detected per week was 6.92. The number of patients detected correctly (true positives) was 5.72, whereas the number of false positives was 11.44 in the training dataset (mean population per week = 73.06). For cutaneous syndrome group, the optimal probability threshold was 0.0442 (Figure 2). The corresponding expected number of infected patients detected per week was 1.65. The number of true positives was 1.30, and the number of false positives was 2.60. For gastrointestinal syndrome group, the number of false positives was high, even at a sensitivity of 75%. It was not possible to obtain a probability with a false positives/true positives ratio of 2, and no optimal threshold could be determined for gastrointestinal syndrome group. The best ratio obtained for a range of sensitivity between 75% and 100% was 22. The corresponding probability threshold was 0.0115 (Figure 2). The expected number of infected patients per week was 0.80; at this threshold, 0.60 were correctly detected, but 13.23 false positives were also identified.

### Evaluation of detection algorithms

#### Cross-validation procedure

Differences between the distributions of individual probabilities in the training and test datasets were not statistically significant for the three syndrome groups (t-test p-values were 0.346, 0.888, and 0.535, respectively, for respiratory, cutaneous and gastrointestinal syndrome groups).

ROC curves of the training and test datasets were compared (Figure 3). Areas under the ROC curves for the detection of patients with SGRS were 0.8977 (95% CI 0.8849–0.9106) in the training dataset and 0.9029 (95% CI 0.8856–0.9201) in the test dataset. For the detection of patients belonging to the cutaneous syndrome group, they were 0.9259 (95% CI 0.8974–0.9543) and 0.9487 (95% CI 0.9152–0.9823), respectively, in the training and test datasets. For the detection of patients belonging to the gastrointestinal syndrome group, they were 0.8668 (95% CI 0.8207–0.9129) and 0.8891 (95% CI 0.8235–0.8547), respectively, in the training and test datasets.

#### Evaluation of the performances of algorithms

The algorithms’ performances were evaluated using the test dataset. Table 4 details the results of algorithm performance for the optimal threshold of probability determined previously with training dataset for a false positive/true positive ratio = 2. Sensitivity is above 75% as it was fixed in the learning phase, and the PPV reflects the fixed false positive/true positive ratio, except for the gastro-intestinal syndrome group, where it was not possible to achieve a ratio of 2 with a sensitivity of 75%. In the gastro-intestinal syndrome group, for each true positive detected and validated, it would be necessary to invalidate 22 false positive detected if this rule of a threshold of a false positive/true positive ratio = 2 had not been set.

**Table 4:** Performances of algorithms in the test dataset

**Syndrome**	**Infected**	**Non-infected**	**Optimal threshold of probability**	**Sensitivity**	**Specificity**	**Positive predictive value**	**Negative predictive value**
				**% (95% CI)**	**% (95% CI)**	**% (95% CI)**	**% (95% CI)**
Respiratory	318	2,950	0.0661	82.70 (78.09–86.70)	82.37 (80.95–83.73)	33.59 (30.28–37.02)	97.79 (97.13–98.33)
Cutaneous	73	3,195	0.0442	78.08 (66.86–86.92)	95.93 (95.19–96.59)	30.48 (23.97–37.62)	99.48 (99.16–99.17)
Gastrointestinal	34	3,234	0.0115	79.41 (62.1–91.3)	81.97 (80.6–83.28)	4.43 (2.94–6.37)	99.74 (99.46–99.89)

## Discussion

The early detection of patients with potentially transmissible infectious diseases, at the beginning of their hospital stay, is an important element in the prevention of nosocomial infections. Such detection is based on the principle of syndromic surveillance, as patients mostly come to EDs with symptoms rather than diagnoses. Moreover, the diagnosis at the time of ED discharge is usually at the stage of hypothesis rather than confirmation. The surveillance system we assessed analyses all available data in computerised ED records, first by automatically processing textual data and then by applying detection algorithms to textual and structured variables in those records.

Our study revealed that syndromic surveillance makes it possible to detect patients with potentially transmissible infectious diseases; sensitivity of detection ranged from 78.08% (cutaneous syndrome group) to 82.70% (SGRS). Detection algorithms have been developed using a common modelling strategy for different syndromes groups. All the variables of the final model presented odds-ratios above 1 except biological examination in biological procedures. Biological examination in biological procedures has odds ratios significantly below 1 in biological procedures (Tables 1, 2 and 3) but the odds ratio is above 1 in clinical notes (Table 1) and in chief complaint (Table 3). The probable explanation is that biological examination mentioned in the biological procedures of the electronic medical records includes systematic biological procedures which are not all specific of the diagnosis procedure of an infection (e.g., blood cells count, C-reactive protein, blood sedimentation). These biological examinations are prescribed for many patients whatever the chief complain (infectious disease or myocardial infection or dehydratation…). Globally this item is then predictive of the absence of infection. Conversely, when a biological examination is explicitly mentioned by the clinician in the chief complaint section or in the clinical note of the electronic medical record, it is probably more often because the biological examination provides relevant information in the diagnostic approach of an infectious disease.

The seasonality was assessed in the regression model, with « month » and « epidemic season » variables. These variables were not significant and were not kept in the final model. This is probably due to the heterogeneity in infectious diseases included in each syndrome group. For example, in the SGRS, there were influenza (seasonal variations) and tuberculosis (no variation according to season).

The thresholds were chosen to obtain the best balance between the ability to detect true positives (sensitivity) and excess workload (false positives), in accordance with the available capacity of the infection-control team at our hospital to address these issues. The threshold is customizable according to the risk of transmission of infection in the hospital where the system will be implemented and also if a different excess workload would be judged acceptable to the infection control team of this hospital.

In evaluating performance, priority was given to high sensitivity, because the goal of the surveillance system was to detect patients with potentially transmissible infectious diseases and thereby allow application of early, suitable, transmission-based precautions. This is why the sensitivity range was set between 75 and 100% to determine the optimal threshold. However, the numbers of true and false positives as a function of sensitivity (Figure 2) revealed that for each syndrome, the number of false positives increased rapidly as sensitivity grew, whereas the detection of true positives did not increase significantly. Improvements could be made that reduce the number of false positives. Among symptoms extracted by textual analysis, one could chronologically distinguish the symptoms that correspond to the patient’s medical history from those that correspond to the current medical situation during the consultation in the ED. For example, in our study, many patients without gastrointestinal infections had diarrhoea among their presenting symptoms in the days before consultation, or chronically, and were identified as being potentially infected. Our extraction system in its current version cannot exclude the symptoms of the medical history, which generates a number of false positives that would be too large for its routine use in the detection of gastrointestinal syndromes. The introduction of semantic analysis should allow association of the symptoms with either the patient’s medical history or the current episode, and should consequently improve the performance of detection for gastrointestinal syndromes.

Our study has some limitations. In particular, the subjects included only patients who were hospitalised after ED consultation. The study did not consider those who were discharged directly after ED consultation, but were potentially contagious to healthcare workers or other patients that they met during their ED stay (e.g., in waiting rooms or elevators). However, our choice to focus exclusively on a hospitalised population was guided by the desire to have a gold standard, namely, validated medical diagnoses in hospital discharge summaries for the purpose of ascertaining infections.

The detection algorithms described here represent overall performance of the automated clinical decision support system being developed in our hospital for syndromic surveillance. This automated clinical decision support processes into two steps: 1) automated extraction of medical terms in the ED record by Urgindex and 2) computation of individual probabilities to be infected using parameters of the logistic final model. The quality of data used for modelling depends also on the quality of the information in the ED electronic record and on UrgIndex performances for automated extraction of the medical terms. UrgIndex recall was 85.8% (95% CI 84.1–87.3), with precision of 79.1% (95% CI 77.3–80.8) [13]. The ability of the system to detect patients with potentially transmissible infectious diseases was susceptible to variation according to healthcare workers’ vocabulary, because automatic UrgIndex processing is based on keyword searches and filters. Repeated revision of detection-tool performance is necessary to adapt the filters to new vocabulary or acronyms in patient records. Analysis of the reasons for lack of detection will allow us to complete different filters, and thus to improve the functionality of the system.

Methods for detecting respiratory, cutaneous and gastrointestinal syndromes from ED records have been extensively reported in the literature. The syndromic surveillance systems described to date mostly use free-text chief complaints as the data source for syndrome detection [5,6,16-20], and automatic text processing to classify chief complaints into syndromes [5,6,16,17,19,20]. However, the sensitivity of respiratory, cutaneous and gastrointestinal syndrome detection has ranged from 43% to 100%, 46.8% to 100%, and 32 to 98.1%, respectively [6,18,21-25]. The variability among the published results can be explained by differences in the syndrome definitions of surveillance systems, divergent detection algorithms and diversity in the data analysed, whereas the performances reported in our study varied according to the syndromes considered and the detection algorithms being tested. The sensitivities we evaluated fell within the intervals described in earlier publications about such systems. However, Elkin et al. demonstrated the superior accuracy of using whole encounter notes, instead of only chief complaints, to detect patients with influenza; those authors processed data similar to those used in our study [26]. At the fixed specificity of 40%, the sensitivity of using whole encounter notes was 89.0%. Consistent with our results, specificities over 94% have been reported in the literature for the three syndromes we studied.

A future stage of our project will involve applying these algorithms to patients who visit the ED, but are not hospitalised thereafter, for diseases such as measles or influenza-like illness. These patients need to be detected as soon as possible, to modify their care in EDs appropriately: isolation in dedicated waiting rooms, rapid care and limitation of cross-contact between potentially infected patients and those who are not infected. The strategy for building the detection algorithms is transferrable to other data environments where explicit outcome labelling such as the final diagnosis is available. The method could be applied to other medical topics where automated detection methods are useful.

## Conclusions

Syndromic algorithms for detecting patients with potentially transmissible infectious diseases based on computerised ED records perform reasonably well for SGRS and cutaneous syndrome group, with an acceptable balance between sensitivity and excess workload associated with the validation of false positives. By contrast, the algorithms tested here for gastrointestinal syndrome group do not permit their routine application. In this study, the threshold of detection was parameterized in order to detect one transmissible disease diagnosis for every three patients flagged (false positive/true positive ratio = 2), if this rule catches enough (sensitivity>75%) of the cases of interest. This threshold was not reached for the gastrointestinal syndrome group.

Algorithms to detect patients with potentially transmissible infectious respiratory or cutaneous infections need to be assessed in a prospective syndromic surveillance system. Based on the results of our feasibility study, we are developing as part of a new collaborative research project with two industrial partners, a solution that can be integrated into the hospital information system and implemented for routine use (SYNODOS project) [27]. This development will permit prospective evaluation. This system will help infection-control practitioners to confirm that transmission-based precautions are implemented as soon as patients come in contact with healthcare facilities to prevent the transmission of infectious diseases.

## Abbreviations

ED: Emergency department; NPV: Negative predictive value; PPV: Positive predictive value; SGRS: Syndrome group of the respiratory system.

## Competing interests

The authors declare that they have no competing interests.

## Authors’ contributions

SGC and MHM conceived the study. SG undertook the analysis to build and assess the algorithms. QG designed and constructed the text-processing application and improved it when necessary. QG worked on algorithms to group codes of medical terminologies according to concept unique identifier of the UMLS and assess the performances. ALM participated in data collection with the DMU’s data warehouse. SG, VP, CR and MHM evaluated and determined which infectious diseases were important to detect. CR extracted hospitalisation discharge diagnostic codes to constitute the gold standard. SD performed ECMT algorithms. SG drafted the manuscript and MHM revised it. All authors have read, revised and approved the final version of the manuscript.

## Pre-publication history

The pre-publication history for this paper can be accessed here:

http://www.biomedcentral.com/1472-6947/13/101/prepub
